# Out-of-hours demand for GP care and emergency services: patients' choices and referrals by general practitioners and ambulance services

**DOI:** 10.1186/1471-2296-8-46

**Published:** 2007-08-01

**Authors:** Eric P Moll van Charante, Pauline CE van Steenwijk-Opdam, Patrick JE Bindels

**Affiliations:** 1 Department of General Practice, Academic Medical Centre, University of Amsterdam, Meibergdreef 15, 1105 AZ Amsterdam, The Netherlands; 2 Primary Health Care Group Almere, Randstad 22-01, 1316 BN Almere, The Netherlands

## Abstract

**Background:**

Over the last five years, Dutch provision of out-of-hours primary health care has shifted from practice-based services towards large-scale general practitioner (GP) cooperatives. Only few population-based studies have been performed to assess the out-of-hours demand for GP and emergency care, including the referral patterns to the Accident and Emergency Department (AED) by GPs and ambulance services.

**Method:**

During two four-month periods (five-year interval), a prospective cross-sectional study was performed for a Dutch population of 62,000 people. Data were collected on all patient contacts with one GP cooperative and three AEDs bordering the region.

**Results:**

Overall, GPs handled 88% of all out-of-hours contacts (275/1000 inhabitants/year), while the AED dealt with the remaining 12% of contacts (38/1000 inhabitants/year). Within the AED, the self-referrals represented a substantial number of contacts (43%), although within the total out-of-hours demand they only represented 5% of all contacts. Self-referrals were predominantly young adult males presenting with an injury, nineteen percent of whom had a fracture. Compared to self-referrals, patients who were referred by the GP or brought in by the ambulance services were generally older and were more frequently admitted for both injury and non-injury (p < 0.01 for all differences).

**Conclusion:**

The GP cooperative deals with the large majority of out-of-hours problems presented. Within the total demand, self-referrals constitute a stable, yet small group of patients, many of whom seem to have made a reasonable choice to attend the AED. The GPs and the ambulance services appear to be effectively selecting the problems that are presented to the AED.

## Background

Following the UK and Denmark, over the last five years, Dutch provision of out-of-hours primary health care has shifted from practice-based services towards large-scale general practitioner (GP) cooperatives [1,2]. There are currently more than 130 GP cooperatives in the Netherlands, generally with 40 to 120 full-time participating GPs, which cover over 90% of the entire Dutch population and serve between 50,000 and 500,000 people. Most GP cooperatives are known to lie in close proximity of the hospital. Although most GP cooperatives operate independently from the hospital, recently, some have decided to integrate with the local Accident & Emergency Department (AED), to form one out-of-hours emergency centre [3]. One of the motives for this reorganisation was to prevent patients from self-referring themselves directly to the AED without first consulting the GP cooperative. Some authors have pointed out, that many of these so called self-referrals present with minor problems that can also be treated by a GP [4,5]. This has led Dutch health policy makers to believe that integration of all out-of-hours services using one triage system will offer a chance to improve the efficiency and quality of care at a lower cost. Likewise, patient organisations have pointed out that patients find it increasingly difficult to determine to whom they should turn with their out-of-hours demand: the GP cooperative, the AED or the ambulance services.

Before major reorganisations are to take place, comprehensive data on overall out-of-hours care utilization should be provided, based on well defined populations. Interestingly, so far, only few studies have attempted to do so [3,6-11].

In this paper we describe the out-of-hours demand for a Dutch population of 62,000 people. The objective of this study is (1) to determine the out-of-hours patterns of use of general practice and A&E services; (2) to compare AED visits by self-referrals, patients referred by the GP cooperative, and patients brought in by the ambulance services.

## Methods

### Setting

The GP cooperative in the coastal city of IJmuiden, the Netherlands, took part in the study. Within a well defined area (municipality of Velsen) it serves a population of around 62,000 people with a total of 25 GPs and eight nurses. The age and sex distribution of the population studied appears to be fairly similar to that of the Dutch population (Table 5).

During out-of-hours, all staff members have access to all electronic medical records for all GP practices (all in- and out-of-hours contacts). The GP cooperative operates from 5 pm to 8 am from Monday to Friday and 24 hours during the weekends. Apart from 11 pm to 8 am when only one GP is on call, two GPs work alongside, one making home visits and one taking care of centre consultations or telephone calls. They are supported by one nurse who receives, assesses, and manages all incoming calls as described elsewhere [12]. She has access to a broad set of guidelines for most acute problems that was developed by the Dutch College of General Practitioners.

The service is located in the former AED of a small district hospital that had to close in 1996 and was subsequently used to harbour the GP cooperative. The population is served by three AEDs bordering the region.

### Subjects and data collection

Between 1 November and 1 March 1997–8 and 2002–3 (two four-month periods), all incoming calls were registered by the telephone nurse. The data-collection was repeated after a somewhat arbitrary period of five years, because GPs were concerned that once the cooperative had become more widely known to the public, increasing numbers of patients would make use of its service. Contact information was entered on a specially prepared data collection sheet. It was completed by the nurses (advice alone) or GPs (all other contacts) and was used to collect demographic data, presented problems (up to a maximum of three), diagnosis (only one, made by GP) and management (by nurse or GP). The International Classification of Primary Care (ICPC) was used to code the presented problem(s), the diagnosis and management [13]. Coding of all contacts was performed by two GP trainees and in case of uncertainty or dispute by an experienced GP who made the final decision. Passers-by from other regions were excluded.

For the same periods of time and population, a similar, retrospective data collection and coding took place using the hospital records for all patients from the population of Velsen who contacted one of the three AEDs. Patients who were referred to the AED after an initial contact with the GP cooperative were also analysed. If these patients had not shown up at one of the AEDs studied, after approximately six months, their electronic medical records were checked for AED reports to locate any other hospitals that were visited after the out-of-hours GP referral.

Similar to Brogan et al., annual rates were estimated by calculating the number of contacts over the study during each weekday evening and night and during each 24 hour period of weekends and bank holidays, and multiplying by 255 weekday evenings/nights and by 110 weekend or bank holiday 24 hour periods [8].

Main outcome measures were: (1) overall contact rates and characteristics of all patient groups contacting the GP cooperative or AED (both periods combined); (2) differences in follow up management between different patient groups contacting the AED (self-referrals, referred by GP and brought in by ambulance services).

The data were analysed in SPSS, version 12.0. Pearson's χ^2 ^test was used to test for differences in two by two tables, using a level of significance of p < 0.05.

## Results

### Patients' contacts with out-of-hours services

During the two four month periods within the study population there were 11,375 contacts with the GP cooperative (87.8%) and 1,584 contacts with the AED (12.2%)(Table 1). Between the two study periods the out-of-hours demand appeared to be fairly stable, showing no significant differences in overall demand for the GP cooperative or emergency services. The total rate of out-of-hours contacts for the population studied was 313 per 1000 inhabitants per year (275 and 38 contacts per 1000 inhabitants for the GP cooperative and AED respectively).

**Table 1:** Total out-of-hours demand in two periods of four months (Nov-Feb 1997/8 and 2002/3)

	1997/8		2002/3		Both periods combined	
	n (%)	n/1000/yr	n (%)	n/1000/yr	n (%)	n/1000/yr

**Contact with GP cooperative**	**5828 (88.2)**	**282.8**	**5547 (87.3)**	**267.1**	**11375 (87.8)**	**274.6**
Telephone advice	2446 (37.0)	118.7	2295 (36.1)	110.5	4741 (36.6)	114.4
Centre consultation	2786 (42.2)	135.2	2622 (41.3)	126.3	5408 (41.7)	130.5
Home visit	596 (9.0)	28.9	626 (9.9)	30.1	1222 (9.4)	29.5
						
**Contact with AED**	**776 (11.8)**	**37.7**	**808 (12.7)**	**38.9**	**1584 (12.2)**	**38.2**
Referred by GP	326 (4.9)	15.8	338 (5.3)	16.3	664 (5.1)	16.0
Self-referral	333 (5.0)	16.2	344 (5.4)	16.6	677 (5.2)	16.3
Via ambulance services	109 (1.7)	5.3	115 (1.8)	5.5	224 (1.7)	5.4
Other (e.g. police/unspecified)	8 (0.1)	0.4	11 (0.2)	0.5	19 (0.1)	0.5
						
**Total number of out-of-hours contacts**	**6604 (100.0)**	**320.4**	**6355 (100.0)**	**306.1**	**12959 (100.0)**	**312.8**

Of those patients who contacted the GP cooperative in both periods combined (n = 11,375), 4741 (41.7%) received a telephone advice, 5408 (47.5%) a centre consultation, and 1222 (10.7%) a home visit. Overall, around 10% of the patients visited the GP cooperative without calling the service in advance. The rate of calls resulting in a nurse telephone advice alone rose from 21.1% in the first period to 31.9% in the second period (p < 0.001), leading to a reciprocal decrease in telephone consultations by the GPs from 20.9% to 9.5%.

Of those patients who contacted the AED in both periods combined (n = 1584), self-referrals represented a substantial number of contacts (42.7%; 677/1584), however, within the total out-of-hours demand, they represented 5.2% (677/12959) of all contacts only.

Overall, women had more contacts with the GP cooperative while men accounted for a higher proportion of those patients who contacted the AED (Table 2). Children under five years accounted for more than three times the proportion of consultations at the GP cooperative compared with the AED, while young adults accounted for a high proportion of those attending the AED.

**Table 2:** Characteristics of patients presenting at the GP cooperative and AED (self-referrals and via ambulance service)

	GP cooperative		AED self-referrals		AED via ambulance		Total demand		
	n	%	n	%	n	%	n	%	n/1000/yr

Male sex	5313	47	360	53	132	59	5819	47	140.5
Public insurance	7885	69	465	69	164	73	8527	69	205.8
									
Age groups									
0–4	2140	19	39	6	5	2	2184	18	52.7
5–14	1134	10	68	10	3	1	1205	10	29.1
15–24	1000	9	167	25	22	10	1189	10	28.7
25–44	2924	26	233	34	45	20	3202	26	77.3
45–64	1803	16	116	17	53	24	1972	16	47.6
≥65	2374	21	54	8	96	43	2524	21	60.9
									
Day (8 AM – 5 PM)	4748	42	218	32	52	23	5022	41	121.2
Evening (5 PM – 11 PM)	5042	44	370	55	118	53	5543	45	133.8
Night (11 PM – 8 AM)	1537	14	89	13	54	24	1682	14	40.6
									
Total	11375	100	677	100	224	100	12276	100	296.3

Both out-of-hours periods combined (without GP referrals).

### Problems presenting to GP cooperative and AEDs

**Figure 1:**
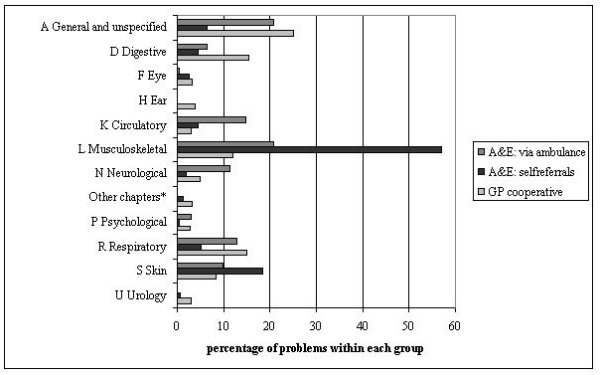
**ICPC chapter of all presenting problems from patients contacting the GP or AED (self-referrals or via ambulance services)**. for both periods combined. * Chapters B,T,W,X,Y and Z combined.

Patients contacting the GP cooperative mainly presented with general and unspecified problems (25.1%), followed by digestive (15.3%), respiratory (15.1%) and musculoskeletal problems (12.0%) (Figure 1). Self-referrals at the AEDs predominantly presented with musculoskeletal (57.0%) or skin problems (18.5%), while those who were brought in by the ambulance services presented general and unspecified (20.8%), musculoskeletal (20.8%) or circulatory (14.7%) problems. The top ten problems that were encountered showed clear differences between the groups studied (Table 3). GP cooperatives were confronted with many questions regarding the medication (request for prescription or advice on medication use), while complaints like fever, cough, vomiting, shortness of breath and earache were also frequently reported. Self-referrals at the AED mainly presented with injury of the extremities and skin lacerations. Patients who came via the ambulance services frequently showed non-traumatic problems (chest pain, syncope, shortness of breath) as well as traumatic problems that were often related to street accidents (skin lacerations, head injury, general injury).

**Table 3:** Ten most frequently presented problems for GP cooperative, self-referrals (AED) and patients brought in by the ambulance services

GP cooperative	n	%	AED: self-referrals	n	%	AED: ambulance services	n	%
1. Fever	1549	7.9	1. Skin laceration/wound	90	12.4	1. Chest pain	29	10.9
2. Request for prescription	971	5.0	2. Hand/fingers	72	9.9	2. Syncope	29	10.9
3. Cough	863	4.4	3. Ankle	71	9.8	3. Shortness of breath	20	7.5
4. Vomiting	706	3.6	4. Wrist	69	9.5	4. Skin laceration	17	6.4
5. Shortness of breath	649	3.3	5. Knee	67	9.2	5. Head injury	12	4.5
6. Earache	625	3.2	6. Foot	46	6.3	6. Coma	11	4.2
7. Advice regarding medication	512	2.6	7. Shortness of breath	19	2.6	7. General injury	10	3.8
8. Skin laceration	471	2.4	8. Leg/thigh	18	2.5	8. Hip	10	3.8
9. Diarrhoea	451	2.3	9. Chest pain	16	2.2	9. Paresis/paralysis	8	3.0
10. Generalised abdominal pain	449	2.3	10. Arm	14	1.9	10. Leg/thigh	7	2.6
								
Total	19562	100.0		733	100.0		265	100.0

Both periods of four months combined. Totals from all presented problems (up to 3 per patient).

### Referrals to the AED

In total, the GPs referred 7.5% of the patients to the AED (853/11375), although only 5.8% (664/11375) eventually arrived in one of the three AEDs (Table 1). Further analysis from the electronic medical records revealed that the remaining 179 patients who were lost to follow up had either travelled to hospitals farther away (97/179, 54.2%) or never seemed to have gone at all (69/179, 38.5%), while 13 cases could not be retrieved (7.3%).

Presentations that were most likely to be associated with a referral to hospital were: chest pain, 120/292 (41.1%); shortness of breath, 116/642 (18.1%); and localised abdominal pain, 45/298 (15.1%). Of all 1972 injuries that were presented to the GP, 233 (11.8%) were referred to the hospital, while of all 9315 non-injuries only 619 (6.6%) patients were referred (difference -5.2%, 95% CI -6.7 to -3.6%).

### AED: self-referrals, patients referred by the GP and by the ambulance services (Table 4)

**Table 4:** AED: self-referrals, patients referred by the GP and patients brought in by the ambulance services

	Self-referral	Via GP	Via ambulance	Total
	n (%)	n (%)	n (%)	n (%)

Male	360 (53.2)	349 (52.6)	132 (58.9)	841 (53.7)
Mean age (sd)	32.7 (19.7)	47.0 (28.8)	55.6 (23.7)	42.0 (26.0)
				
**Injury**	**537 (80.1)**	**226 (35.0)**	**92 (41.4)**	**855 (55.6)**
*Fracture*	*103 (19.2)*	*93 (41.2)*	*25 (27.2)*	*221 (25.8)*
Admission	17 (3.2)	22 (9.7)	24 (26.1)	63 (7.4)
Appointment outpatient clinic	173 (32.2)	117 (51.8)	30 (32.6)	320 (37.4)
Referral to own GP	347 (64.6)	87 (38.5)	38 (41.3)	472 (55.2)
				
**Non-injury**	**133 (19.9)**	**420 (65.0)**	**130 (58.6)**	**683 (44.4)**
Admission	47 (35.3)	294 (70.0)	98 (75.4)	439 (62.6)
Appointment outpatient clinic	43 (32.3)	67 (16.0)	12 (9.2)	140 (20.0)
Referral to own GP	43 (32.3)	59 (14.0)	20 (15.4)	122 (17.4)
				
**Total**	**677 (43.3)**	**664 (42.4)**	**224 (14.3)**	**1565 (100.0)**

Valid %. Due to missing numbers (up to 27), columns do not always add up to their totals. Both periods of four months combined.

Self-referrals had a lower mean age (33 yrs) than those who were referred by the GP (47 yrs) or the ambulance services (56 yrs) (p < 0.001 for all differences).

Most of the self-referrals to the AED presented with an injury (80.1%). This percentage was substantially lower among patients who were referred by the GP (35.0%) or the ambulance services (41.4%). Within the injury group, 19.2% of the self-referrals were found to have a fracture, compared to 41.2% of the GP referrals (p_adj_* < 0.001) and to 27.2% of ambulance service referrals (p_adj _= 0.091). Similarly, fewer self-referrals with an injury were admitted (3.2%) than those who were referred by the GP (9.7%)(p_adj _= 0.002) or the ambulance services (26.1%)(p_adj _< 0.001). On the other hand, self-referrals were referred back to their GP (or received no specific advice to return at all) more often (64.6%) than patients who had been referred by the GP cooperative (38.5%)(p_adj _= 0.003) or the ambulance services (41.3%)(p_adj _= 0.009).

Although the percentage of non-injury among self-referrals was low (19.9%), the admission rate among these patients was substantially higher (35.3%) than among the patients with an injury (3.2%). Nevertheless, the admission rate among non-injury patients who had been referred by the GP was almost twice as high (70.0%)(p_adj _< 0.001) and even higher among those who had been brought in by the ambulance services (75.4%)(p_adj _< 0.001). Likewise, self-referrals with a non-injury were twice as likely to be referred back to their GP as the other two patient groups with non-injury (p_adj _< 0.001).

* p-value adjusted for age, distance to the GP cooperative, and type of problem (ICPC chapter). No effect on mode of care choice was found for sex, type of insurance, social deprivation or time of the day.

## Discussion

This study shows that the GP cooperative is the main provider of out-of-hours care for the population studied. Within the group of all patients who contacted the AED, self-referrals constituted a large group, although they only represented a small percentage of all out-of-hours demand. With nineteen percent fractures among the patients with injury and one third of non-injury patients being admitted to the hospital, a substantial part of self-referrals appeared to have made a reasonable choice to attend the AED. Finally, compared to the self-referrals, both the GP and the ambulance services appear to be an adequate filter to the AED services, referring patients with more fractures and resulting in more hospital admissions.

This study was based on a relatively small population and therefore the results may not be generalisable to other regions. Although the population studied appeared similar to that of the Netherlands in terms of age-sex structure (Table 5), people from the respective age-groups for 15–44 and ≥65 years may have been slightly under- and overrepresented, leading to differences in overall demand from these groups. Furthermore, the proportion of self-referrals within the group of all patients who visited the three AEDs combined was derived from the contacts of the population of Velsen only. It is therefore unknown whether this was also representative for the other populations that these AEDs were serving. Hence, the conclusion that further integration between the GP cooperative and AED may not be effective refers to the population of Velsen only. Another limitation of the study was the relatively high percentage of patients visiting the GP cooperative without calling in advance (10%). Many of these patients presented with an injury (54%), suggesting some similarity with AED self-referrals (the GP cooperative being located in a former AED) and perhaps resulting in an underestimation of the population's overall AED self-referral rate. Nevertheless, even if most of these patients would have attended the AED, they still represent a small part of the overall demand only.

The coding of presenting symptoms to the AED took place using hospital records rather than the data collection sheets that were used in the GP cooperative. Even though the same coding methods were used for both settings, it is not clear whether the order in which the complaints were written down was similar to the order in which they were mentioned. Furthermore, it is uncertain whether the ICPC is valid for problems presented at the AED. Therefore, coding differences between both setting may have occurred.

Finally, the Winter period could have yielded a higher out-of-hours demand, although this effect is likely to have been small [14,15].

Although GPs had been concerned that the cooperative would face an increasing demand once the service became more widely known to the public, no such increase was observed, which is consistent with findings from a study in another Dutch city surveying five consecutive years of out-of-hours demand.

Our study findings appear to be similar to those found in a few other population based studies in the Netherlands [3,11]. Nevertheless, one study in the city of Amsterdam found a substantially lower contact rate with the GP services (171/1000/yr) and higher contact rate with the AED (170/1000/yr). Even larger differences in demand for GP care were found between studies from the UK, Ireland, Denmark and Finland (ranging from 130/1000/yr in England to 533/1000/yr in Finland) [14,16-18]. International comparisons should be interpreted cautiously because of varying definitions of the out of hours period and differences in health service organisation. Salisbury et al. found that the variation in call rates between different British cooperatives could not be accounted for by local demographic features (age structure, deprivation, and rurality) [14]. Finally, although no literature review has been performed yet, self-referral rates to the hospital too appear to vary substantially across some European countries (ranging from 57/1000/yr in one British study to 190/1000/yr in Denmark) [8,10,19,20] Perhaps the enormous variation is in part explained by differences in the effectiveness of the gatekeeper. Boerma and others have shown, that while as a rule patients need a GP referral to make use of hospital services, all gatekeeping systems make an exception for emergencies that can be presented directly to the AED [21]. This important leakage of gatekeeping systems may lead to variation in AED use, especially since the (perceived) availability of GP services varies from country to country [22-24].

Various authors have expressed their concern about the overcrowding of AEDs as a result of high numbers of self-referrals [25,26]. There has been much debate on how to redirect these allegedly 'inappropriate attenders' to the GP. However, without a clear definition of what constitutes an 'inappropriate attender', it seems not surprising that a wide variation (6–80%) was found in the literature [27], and that others have cast doubt on the usefulness of the term itself [28]. In our study, with nineteen percent fractures among the injuries and thirty-five percent admissions among the non-injuries, at least the self-referrals emerge as a self-selected group with a severity level that appears to be higher than patients calling the GP cooperative, but still lower than those who were referred by the GP [29]. Also, in part, the care seems complementary: while 80% of the self-referrals presented with an injury, GPs also referred significantly more injury patients (11.6%) to the AED than patients without an injury (6.6%). Overall, many patients may have made reasonable choices when deciding which service to contact [9,30]. Nevertheless, compared to the self-referrals, the GP and ambulance services provide an effective patient filter to the AEDs.

If GP cooperatives and AEDs are to further integrate their services, more research is needed on out-of-hours demand. Patient characteristics and their motives to attend to an AED as a self-referral or call the emergency services rather than contacting a GP cooperative need further elucidation before radical organisational changes are to be carried through [9,31-33]. Also, more insight is needed into the triage activities of the regional ambulance services, both on the telephone and during field assessments, as they appear to overlap with both GP and AED services [34].

From the patients' perspective, having one national or regional emergency number and one out-of-hours emergency service for all problems presented may seem an obvious development. Although some studies have indicated that GPs working within the AED handle self-referrals equally safe with fewer use of resources [5,35], it is unclear whether integration of all services would become more efficient in terms of professional care and costs, as this may be more dependent on the size of the population the cooperative covers than the way the GP cooperative is organised, i.e. separated or integrated [36]. Moreover, except for the major cities, self-referrals not only represent a relatively small group in the total out-of-hours demand, many of these patients may have made a reasonable choice for AED services [9,30].

It is not unlikely that different, regional models of integration could evolve from local patient demand. In the Netherlands, a few integrated out-of-hours emergency centres are now operational [3], although many GP cooperatives and AEDs prefer to keep the provision as it is and focus on working together more closely.

## Conclusion

GP cooperatives appear to deal with the large majority of all out-of-hours problems presented.

Within the total out-of-hours demand, self-referrals at the AED constitute a small group of patients who, in part, seem to have made a reasonable choice of service. Compared to the self-referrals, GPs and ambulance services appear to make stronger selections of injury and non-injury patients as is indicated by higher percentages of fractures and hospital admissions.

## Competing interests

The author(s) declare that they have no competing interests.

## Authors' contributions

EMC planned the design of the study, collected most of the data, took part in the analysis and led the writing of the paper. PSO contributed to the writing of the paper and drafted the figure. PB contributed to the design of the study, the analysis and to the writing of the paper.

All authors read and approved the final version of the manuscript.

**Table 5:** Age and sex of patients contacting the out-of-hours services, compared with the population of Velsen and the Dutch population.

	All out-of-hours contacts				Population of Velsen*				Population of the Netherlands*			
	1997–8		2002–3		1997–8		2002–3		1997–8		2002–3	

	n	%	n	%	n	%	n	%	n	%	n	%

Sex												
Male	2975	47.4	2830	47.1	29877	49.1	30627	49.2	7740074	49.4	8015471	49.5
Female	3286	52.4	3168	52.7	31013	50.9	31664	50.8	7914118	50.6	8177101	50.5
Missing	9	<1	8	<1								
												
Age bands (years)												
0–4	1244	19.8	940	15.7	4251	7.0	4086	6.6	969367	6.2	1022613	6.3
5–14	600	9.6	605	10.1	7247	11.9	8195	13.2	1913563	12.2	1987475	12.3
15–44	2287	36.5	2104	35.0	25353	41.6	24325	39.1	6951802	44.4	6858760	42.4
45–64	967	15.4	1005	16.7	13841	22.7	15360	24.7	3709741	23.7	4103268	25.3
≥65	1172	18.7	1352	22.5	10198	16.7	10325	16.6	2109719	13.5	2220456	13.7
Total	6270	100.0	6006	100.0	60890	100.0	62291	100.0	15654192	100.0	16192572	100.0

*1 January 1998 and 1 January 2003

The data provided represent demographic characteristics of patients contacting the out-of-hours services in the municipality of Velsen (study data), the total population of Velsen (data from the local council of Velsen) and the total Dutch population (data from the Office of National Statistics (CBS)).

## Pre-publication history

The pre-publication history for this paper can be accessed here:


